# Clonal Distribution and Intratumor Heterogeneity of the TCR Repertoire in Papillary Thyroid Cancer With or Without Coexistent Hashimoto’s Thyroiditis

**DOI:** 10.3389/fimmu.2022.821601

**Published:** 2022-06-03

**Authors:** Likun Cui, Chaoting Zhang, Huirong Ding, Dongdong Feng, Hongying Huang, Zheming Lu, Baoguo Liu

**Affiliations:** ^1^ Key Laboratory of Carcinogenesis and Translational Research (Ministry of Education/Beijing), Department of Head & Neck Surgery, Peking University Cancer Hospital & Institute, Beijing, China; ^2^ Key Laboratory of Carcinogenesis and Translational Research (Ministry of Education/Beijing), Laboratory of Biochemistry and Molecular Biology, Peking University Cancer Hospital & Institute, Beijing, China; ^3^ Key Laboratory of Carcinogenesis and Translational Research (Ministry of Education/Beijing), Core Laboratory, Peking University Cancer Hospital & Institute, Beijing, China; ^4^ Department of Head and Neck Surgery, Center of Otolaryngology Head and Neck Surgery, Affiliated People’s Hospital of Hangzhou Medical College, Zhejiang Provincial People’s Hospital, Hangzhou, China; ^5^ Department of Pathology, New York University Langone Medical Center, New York, NY, United States

**Keywords:** T cell receptor, intratumor heterogeneity, papillary thyroid cancer, Hashimoto’s thyroiditis, immune repertoire

## Abstract

The intratumor heterogeneity (ITH) of the amount and TCR repertoires of tumor infiltrating lymphocytes (TILs) in PTC with and without coexistent Hashimoto’s thyroiditis (HT) are unclear. Here, we investigated the amount of T cells in tumor and corresponding normal tissues by immunohistochemical staining on 80 tumor samples and 40 normal samples from 40 patients. The immune repertoire of T cells was identified on 24 tumor samples and 12 normal samples from 12 patients using TCR high-throughput sequencing. The results demonstrated that the numbers of CD3+, CD4+ and CD8+ T cells in PTC without coexistent HT (PTC-WO) were significantly lower than those in PTC with existing HT (PTC-W). In PTC-W, the density of CD4+ TILs were generally higher when compared with CD8+ TILs. Furthermore, we found that the numbers of CD3+ T cells and their CD4+, CD8+ subtypes in tumor samples were generally higher than those in normal tissue in PTC-WO and moreover, the number of CD3+ T cells was negatively associated with TCR clonality in PTC-WO. In addition, although ITH of the TCR repertoire truly existed in PTC-W and PTC-WO, the TCR repertoires between distinct regions of the non-adjacent tumor foci were presented with a higher degree of similarity than those between tumor and matched normal tissue in PTC-WO, yet the similarity of intratumor repertoires was not significantly higher than those between tumor and corresponding normal samples in PTC-W. This research comprehensively delineated the quantity and TCR repertoire ITH of T cells in PTC-W and PTC-WO, suggesting that TILs might be reactive to tumor antigens in PTC-WO. Moreover, multiregion biopsies should be performed to precisely identify the immune background in PTC-W and PTC-WO.

## Introduction

Papillary thyroid cancer (PTC) is the most frequent histologic type of differentiated thyroid cancer, and its morbidity increases rapidly worldwide (1). Approximately 23% (range, 5% to 85%) of PTC coexists with Hashimoto thyroiditis (HT) which is the most prevalent autoimmune disease and is characterized by a local immune response involving T and B lymphocytic infiltration of the thyroid tissue, accompanied by a humoral immune response leading to the elevation of thyroid-specific antibodies (2, 3).

Intratumor heterogeneity (ITH) influences the accurate diagnosis, effective treatment, and personal prognosis (4, 5). Therefore, to comprehensively identify the ITH contributed not only by neoplastic cells, but also by nontumor cells in PTC with coexistent HT (PTC-W) and PTC without coexistent HT (PTC-WO) seems necessary. Of nontumor cells, tumor infiltrating lymphocytes (TILs) run through the antitumor response (6, 7). Previous studies, including ours, primarily focused on the ITH of tumor cells (8) and the number of TILs (9) in PTC. Since TILs could specifically identify tumor antigens determined by T cell receptors (TCR), it is necessary to systematically and comprehensively validate the ITH of the amount and TCR repertoire of TILs in PTC-W and PTC-WO.

The T cell diversity is the result of somatic recombination of TCRα and β genes (10, 11). Different from TCRα alleles, which could both functionally recombined in a proportion of T cells, TCRβ alleles are determined strictly allelic exclusion (12). Therefore, the TCRβ chain can be regarded as a straightforward substitute to study the TCR repertoire. Independent somatic rearrangements of variable (V), diversity (D) and joining (J) genes, as well as non-templated ligation in the TCRβ chain can produce the highly diverse complementary determining region 3 (CDR3) that can be a proxy for each T cell clone. Therefore, high-throughput sequencing of TCR β CDR3 alone can directly reflect the T cell repertoire to a large extent (13, 14).

To assess the spatial heterogeneity of TILs in PTC-W and PTC-WO, we systematically evaluated the amount and TCR repertoires of T cells in two distinct tumor regions as well as the matched adjacent normal tissue. The purpose of this study was to: (a) identify the amount and TCRβ repertoires of T cells in the tumor and the adjacent normal tissue; and (b) test whether the intratumoral TCRβ repertoire was spatially heterogeneous in PTC-W and PTC-WO.

## Materials and Methods

### Patients and Sample Preparation

This study was conducted among 20 patients with PTC-W and 20 patients with PTC-WO at Beijing Cancer Hospital (Beijing, China) from January to May 2021. We collected tumor and matched normal tissue from each patient at the time of surgery. Baseline characters are presented in **Table S1** **,** **S2**. All the participants signed an informed consent approved by the Institutional Review Board of Beijing Cancer Hospital, China.

**Figure 1:**
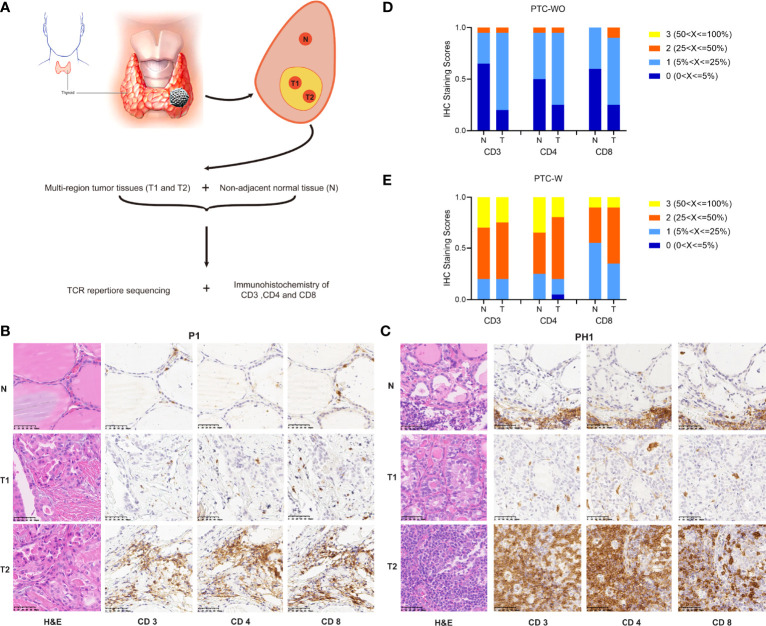
Schematic diagram of sample preparing and comparison of CD3+, CD4+ and CD8+ T cells in PTC with and without HT. **(A)** Schematic diagram of sample collection. **(B, C)** Representative H&E and IHC staining images of CD3+ CD4+ and CD8+ T cells in PTC with and without HT (magnification ×400). **(D, E)** The statistical results of CD3+ CD4+ and CD8+ T cells in PTC with and without HT.

**Table S2** and **Figure 1A** summarized the details of the sample preparation. Briefly, we collected two non-adjacent tumor tissues at the center of the tumor, representing the spatial heterogeneity of different tumor samples. Matched normal tissues were resected from the ipsilateral thyroid lobe and more than 1 cm away from the tumor. Each tumor sample was divided into 2 parts, one for DNA extraction and another for immediate formalin fixation. 60 samples from 20 patients with PTC-W and 60 samples from 20 patients with PTC-WO were used for H&E staining and immunohistochemistry of CD3, CD4, and CD8. The 18 samples from 6 patients with PTC-W and 18 samples from 6 patients with PTC-WO were used for high-throughput sequencing of TCRβ.

### Immunohistochemical Staining

Noncontinuous tumor tissues (tumor 1 and tumor 2), and adjacent normal tissues were fixed in 10% formalin and then embedded in paraffin tissue blocks. Tissues were sectioned at 5 μm and prepared according to standard methodology. For immunohistochemistry staining, the investigated antibodies were mouse monoclonal anti-CD3 (clone LN10; ZSGB-BIO, Beijing, China), mouse monoclonal anti-CD4 (clone UMAB64; ZSGB-BIO, Beijing, China) and rabbit monoclonal anti-CD8 (clone SP16; ZSGB-BIO, Beijing, China). Counterstaining was performed using bluing reagent from ZSGB-BIO; slides were mounted with coverslips and air-dried. The immunostaining images were obtained under a Nikon eclipse 80i microscope (Nikon, Japan) and evaluated by two experienced pathologists in a blinded manner. Five representative fields were selected for each section randomly at 400× magnification. Image-Pro-Plus software (version 6.0; Media Cybernetics, Silver Spring, USA) was used to count the positive cells in each field. The numbers of positive cells were calculated by taking the median. Immunostaining was quantitatively scored based on the percentage of positive cells (0, ≤5%; 1, 6-25%; 2, 26-50%; 3, 51-75%; and 4, >75%).

### High-Throughput Sequencing of TCRβ

Details of TCRβ sequencing were referred to the previous protocols (11, 15, 16). Briefly, the CDR3 gene fragments of the TCRβ chain were amplified through multiplex PCR amplification reaction and sequenced employing the Illumina HiSeq2500 platform (MyGenostics, Beijing, China) from the genomic DNA. The CDR3 nucleic acid sequences of the TCRβ chain was aligned based on the definition established by the International ImMunoGeneTics (IMGT) collaboration (17). Unmatched sequencing reads were filtered out subsequently. A standard algorithm was applied to identify V, D, and J segments contributing to the CDR3 region (17). Since sequences with frameshifts or stop codons are unable to translate into a functional protein, only productive reads were evaluated for followed downstream analyses.

### TCRβ CDR3 Sequencing Analysis

To characterize the diversity of the TCR repertoire for each sample, clonality index were applied which is defined as 1– (Shannon’s entropy)/log2 (number of productive unique reads), ranging from 0 (most diverse) to 1 (least diverse) (18, 19).

We used the Bhattacharyya coefficient based on the frequency and homogeneity of shared TCRβ sequencing reads within two samples, to describe the similarity of the TCRβ repertoire (20):


$$Bhattacharyya=\sum_{i=1}^{n}\sqrt{f(i,1)\times f(i,2)}$$

where n and f(i,1/2) represent for the number and frequency of repeating part of TCR clones within two TCR repertoires. The Bhattacharyya similarity index ranges from 0 to 1, with 0 indicating the scarce of overlap and 1 indicating an identical repertoire between the two subjects.

### Statistical Analysis

A nonparametric Mann-Whitney U test was applied for all statistical analyses. Two-sided *P* values of < 0.05 were considered statistically significant. Spearman’s rho was calculated to characterize the correlation between two variables. All statistical analyses and data visualization were performed in GraphPad Prism V.7.0 (GraphPad Software, California, USA) and R version 4.0.3.

### Data Access

Raw sequencing data were uploaded to the Sequence Read Archive (BioProject No. PRJNA755141).

## Result

### Patient Characteristics

We enrolled 20 patients with PTC-W and 20 patients with PTC-WO, and collected samples of two different tumor regions and corresponding normal tissue for each patient in our study. **Table S1** showed the clinical characteristics of 20 patients with PTC-WO and 20 patients with PTC-W. The clinical characteristics of patient with PTC-WO and PTC-W were comparable, except for preoperative TSH, anti-TPOAb and anti-TGAb. We performed immunohistochemistry analysis of CD3, CD4, and CD8 on all tumor and matched normal samples from 40 patients and TCR high-throughput sequencing on 24 tumor samples and 12 normal samples from 12 patients (**Figure 1A** and **Tables S2**, **S3**).

### Immunohistochemistry Analysis of CD3+, CD4+ and CD8+ T Cells

To delineate the amounts of T cells of normal and tumor tissue in PTC-W and PTC-WO, we evaluated immunohistochemical staining of CD3, CD4 and CD8 on all 80 tumor samples and 40 normal samples from 40 patients in immunohistochemistry analysis. The representative H&E and immunohistochemical staining images demonstrated that CD3, CD4 and CD8 expression was rarely seen and moreover were generally scattered throughout the tumor or normal tissues, but occasionally appeared in clusters in PTC-WO (**Figures 1B**, **S1A**). However, CD3, CD4 and CD8 expression was generally detected across the whole tumor and normal tissues in PTC-W (**Figures 1C**, **S1B**). Meanwhile, we found that the numbers of CD3+ T cells and the CD4+, CD8+ subsets not only in different patients but also in different tumor regions and corresponding normal tissue of the same patient varied greatly in PTC-W and PTC-WO, such as P1 versus P2/P3, and PH1 versus PH2/PH3 (**Figures 1B, C**, **S1A, B**). The stacked bar chart of CD3, CD4 and CD8 staining in 20 patients with PTC-WO and 20 patients with PTC-W showed that the numbers of CD3+, CD4+ and CD8+ T cells of tumor and normal samples in PTC-W were significantly higher than those in PTC-WO (**Figures 1D, E**). Furthermore, we found that the numbers of CD3+, CD4+ and CD8+ T cells in tumor samples were generally higher than those in normal tissue in PTC-WO, but the numbers of CD3+, CD4+ and CD8+ T cells were similar between tumor and normal tissue in PTC-W (**Figures 1D, E**). In addition, we also found that the number of CD4+ T cells was generally higher than that of CD8+ T cells in PTC-W, although the number of CD4+ and CD8+ T cells was similar in PTC-WO (**Figures 1D, E**).

### Clonality Analysis of TCR Repertoire

TCRβ CDR3 regions of infiltrated T cells were amplified and sequenced in two different tumor regions and corresponding adjacent normal tissue of six patients with PTC-W and six patients with PTC-WO. Through sequencing and analysis, a median of 90,264 (IQR 53,688–122,075) and 94,563 (IQR 53,258–156,248) productive CDR3 sequences were distributed with approximately a median of 49,197 (IQR 30,820–74,522) and 57,547 (IQR 29,963–95,471) unique TCRβ clones for PTC-W and PTC-WO, respectively (**Table S3**).

**Figure 2:**
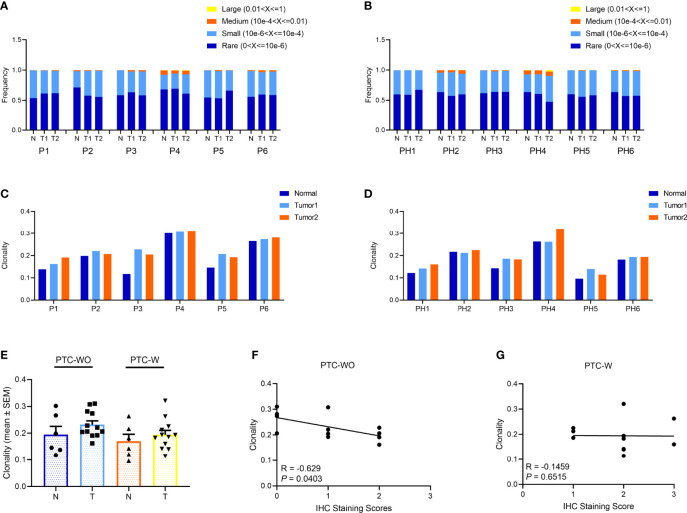
The clonal distribution of the TCR repertoire in PTC with and without HT. **(A, B)** The frequency distribution of TCR clones in PTC with and without HT. **(C, D)** The TCR clonality in PTC with and without HT. **(E)** Comparison of TCR clonality of tumor and normal samples in PTC with and without HT. **(F, G)** Spearman correlation between clonality and IHC staining score of CD3+ T cells in PTC with and without HT.

To delineate the clonal distribution of the TCR repertoire, we primarily evaluated the frequencies and distribution of different TCR clonotypes and found that although the frequencies of large and medium TCR clonotypes from different tumor and normal samples were generally low in PTC-W and PTC-WO, the frequencies of large and medium TCR clonotypes, greatly varied in different patients, such as P1 versus P4, and PH1 versus PH4 (**Figures 2A, B**, **S2**, **S3**). However, for each patient, the distribution of different clonotypes among tumor and normal samples was generally similar in PTC-W and PTC-WO (**Figures 2A, B**). To systematically identify the T-cell repertoire diversity of tumor and normal tissues in PTC-W and PTC-WO, we adopted the metric of clonality as a measure of diversity. Similar to the distribution of different TCR clonotypes, we found that clonality greatly varied from 0.1 to 0.3 among different patients, but for each patient, clonality was generally similar across different tumor and normal samples in PTC-W and PTC-WO, except for P3 (**Figures 2C, D**). In summary, we found that although clonality greatly varied in different patients, clonality in PTC-W was not significantly different from that in PTC-WO and clonality was generally similar between tumor and normal samples in PTC-W and PTC-WO (**Figure 2E**). Furthermore, we evaluated the relationship between the number of CD3+ TILs and TCR clonality, and found that the number of CD3+ TILs was negatively associated with TCR clonality in PTC-WO, but the number of CD3+ TILs was not significantly associated with TCR clonality in PTC-W (**Figures 2F, G**).

### Spatial Heterogeneity of TCR Repertoire

**Figure 3:**
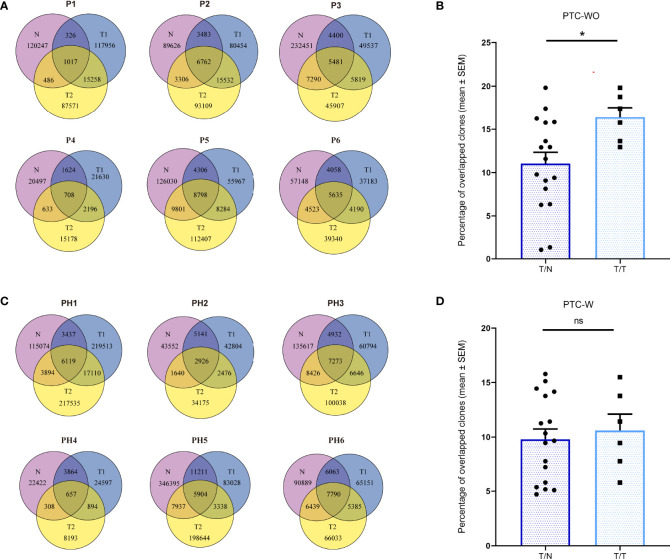
The overlapping proportions of TCR clones across different samples in PTC with and without HT. **(A)** The overlapping proportions of TCR clones among different samples in PTC without HT. **(B)** The overlapping proportions of TCR clones between tumor (T) and normal (N) samples as well as between different tumor samples in each PTC without HT. **(C)** The overlapping proportions of TCR clones among different samples in PTC with HT. **(D)** The overlapping proportions of TCR clones between tumor (T) and normal (N) samples as well as between different tumor samples in each PTC with HT. *P < 0.05; ns, not significant.

To evaluate the spatially heterogeneous degree of the TCR repertoire in PTC-W and PTC-WO, we primarily compared the overlapping TCRβ clones among different tumor samples and corresponding normal samples for each patient. Although overlapping TCR clones between different samples of the same patient with PTC-WO and PTC-W greatly varied, the percentage of overlapping clones between different tumor samples of the same patient with PTC-WO was significantly higher than that between tumor samples and the corresponding normal sample yet the percentage of overlapping clones between different tumor samples of the same patient with PTC-W was similar to that between tumor and matched normal tissue (**Figures 3A–D**).

**Figure 4:**
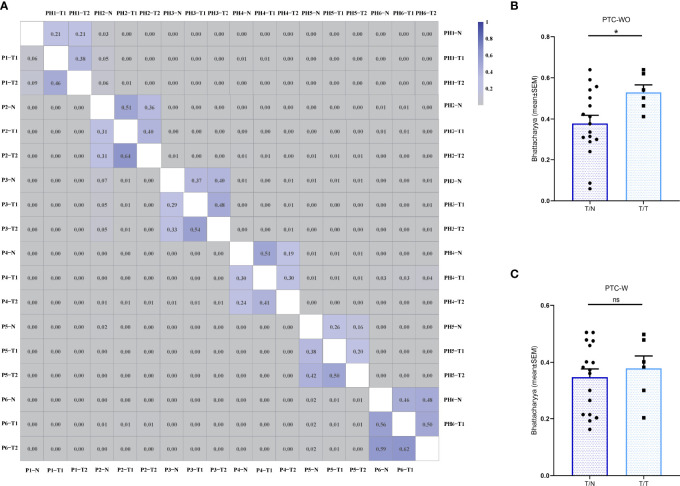
The Bhattacharyya coefficient of the TCR repertoire across different samples in PTC with and without HT. **(A)** The Bhattacharyya coefficient between different samples in PTC with and without HT. The color scales represent for the Bhattacharyya coefficient variation. Two triangles of the heatmap demonstrate the Bhattacharyya coefficient landscape between different samples in PTC with and without HT, respectively. **(B)** The Bhattacharyya coefficient between tumor (T) and normal (N) samples as well as between different tumor samples in each PTC without HT. **(C)** The Bhattacharyya coefficient between tumor (T) and normal (N) samples as well as between different tumor samples in each PTC with HT. *P < 0.05; ns, not significant.

The Bhattacharyya coefficient was adopted to quantitatively evaluate the spatial heterogeneity of the TCR repertoires of different tumor samples and corresponding normal samples, with reference to the abundance and composition of TCR clones. The Bhattacharyya coefficient ranges from 0 to 1 with 0 indicating a completely different TCR repertoire while 1 indicating an identical TCR repertoire. We first found a lack of overlap of the T cell clones among different patients with a Bhattacharyya coefficient approaching 0, as expected (**Figure 4A**). Furthermore, we found that although the Bhattacharyya coefficient between two different samples of the same patient with PTC-WO and PTC-W varied greatly, ranging from 0.06 to 0.64, the Bhattacharyya coefficients between two different tumor samples of the same patient with PTC-WO were significantly higher than those between the tumor sample and corresponding normal sample. In contrast, the Bhattacharyya coefficients between two different tumor samples of the same patient with PTC-W were not significantly higher than those between the tumor sample and corresponding normal sample (**Figures 4A–C**).

## Discussion

Although the amounts of TILs in PTC-W and PTC-WO have been investigated, TCR repertoire analysis of TILs was seldom been reported (21, 22). To our knowledge, we firstly investigated the spatial heterogeneity of the TCR repertoire in PTC with and without existing HT as well as the relationship of the TCR repertoire between PTC with and without existing HT, which will contribute to practical applications for personalized treatment. The spatial heterogeneity of TILs not only affected the diagnosis and prognosis, but also played an important role in selection of immunotherapy methods. The multiregion biopsies are required to comprehensively identify the immune response in PTC-W and PTC-WO, which could provide great insights into the local immune reaction of PTC-W and PTC-WO and increase our understanding of the intratumoral TCR repertoire in solid cancers. In addition, since PTC tended to have a lower mutation burden (23), the limited understanding of tumor-specific neoantigens hampered our efforts to determine specific targets for T cell-mediated immunotherapy. Therefore, we have directly investigated the TCR repertoire as an interpretation for the spatial landscape of immunogenic tumor antigens. The identification of clonally expanded TCRs in distinct tumor regions could also give a hint on selection of tumor-reactive TCRs, which could provide insights into the TILs-mediated adoptive cell therapy for thyroid malignancy.

In PTC, a higher level of ITH not only increases the likelihood of drug resistance and disease recurrence (24), but also has a major impact on the availability of immunotherapy (25). In this study, we found that the numbers of CD3+ T cells and CD4+, CD8+ subsets in different tumor regions and corresponding normal tissue of the same patient varied greatly in PTC-W and PTC-WO and moreover, the Bhattacharyya coefficient between two different samples of the same patient with PTC-WO and PTC-W varied greatly, ranging from 0.06 to 0.64. These findings confirmed that intratumor heterogeneity of the T-cell quantity and TCR repertoire truly existed in both PTC-W and PTC-WO. Multiregion biopsies should be performed to precisely identify the immune background in PTC-W and PTC-WO. Similar to previous findings (26–28), the number of TILs in PTC-W was significantly elevated than that in PTC-WO; moreover, the number of CD4+ TILs were generally higher than the number of CD8+ TILs in PTC-W. Since tumor cells usually expressed MHC class I molecules but not MHC class II molecules, tumor antigens presented by MHC class I molecules could promote the activation and amplification of tumor-reactive CD8+ T cells. Thus, we found that numbers of CD8+ T cells in tumor samples were generally higher than those in normal tissue in PTC-WO and PTC-W, but the numbers of CD4+ T cells were similar in tumor and normal samples. Since thyroglobulin and thyroperoxidase antibodies are the most abundant proteins of the thyroid gland in HT, B cells secreting these antibodies need to be activated by CD4+ Th2 cells. Moreover, recent studies also demonstrated a prominent role of CD4+ Th17 and Treg cells in the proinflammatory microenvironment of HT. Thus, we found that the number of CD4+ T cells was generally higher than that of CD8+ T cells in PTC-W, but the number of CD4+ and CD8+ T cells was similar in PTC-WO.

In this study, we found that the TCR repertoires between distinct regions of the non-adjacent tumor sites were presented with an elevated degree of similarity than those between tumor and matched normal tissue in PTC-WO. Thus, these findings might give a hint that TILs in PTC-WO were significantly different from T cells in corresponding adjacent normal tissue and thus were spatially confined to the tumor microenvironment (TME). In addition, our previous study also found an association between neoantigens and TILs in PTC-WO (18), suggesting that TILs might be reactive to tumor antigens and further clonally amplified. Of course, these findings were only preliminary results due to lack of antigen-specificity information, but in our future study, we will attempt to isolate TILs and corresponding tumor cells in PTC-WO, and then evaluate whether these TILs could identify the corresponding tumor cells through in-vitro co-culture of TILs and autologous tumor cells. These findings might indicate that TIL therapy could be a promising approach for patients with refractory and relapsed PTC-WO.

The quantity of tumor antigens in different tissues could influence the number and diversity of TCR clones. Since the tumor antigens could induce amplification of tumor-reactive T cells, more kinds of tumor antigens could induce more kinds of tumor-reactive T cells, which led to an increase in T-cell number and a decrease in clonality. Thus, we found that the number of CD3+ TILs was negatively associated with TCR clonality in PTC-WO. However, since tumor-reactive TILs could be rare, or numerous T cells targeting multiple thyroid autoantigens due to continuous inflammation were dispersedly infiltrated in the TME of PTC-W, the dispersedly distributed autoreactive T cells masked the effect of tumor-specific immune response and thus the number of CD3+ TILs was not significantly associated with TCR clonality in PTC-W. Of course, the sample size was relatively small and moreover no other evidence confirmed these findings, so this hypothesis is required to be validated.

Since incidence rates of PTC-WO and PTC-W were much higher in female than in male, our study enrolled more female patients (**Supplementary Table S1**). The clinical characteristics of patients with PTC-WO and PTC-W were comparable, except for TSH, anti-TPOAb and anti-TGAb in our study (**Supplementary Table S1**). We also compared the IHC staining scores in the subgroups of female (**Figure S4B**) and male (**Figure S4C**), the result was similar. Due to limited project funding, only the samples of the first six patients with PTC-WO and the first six patients with PTC-W were used for TCR sequencing. By the square, all twelve patients were female due to higher incidence rate of PTC in female. Therefore, TCR repertoire analysis was only conducted in female with PTC-WO and PTC-W. Although IHC staining scores of CD3+, CD4+ and CD8+ T cells were similar in male and female with PTC, relationship of TCR repertoire between male and female with PTC was unknown. Therefore, it is essential to evaluate TCR repertoire in male patient with PTC-WO and PTC-W in the future study. In addition, although all patients with PTC-W were firstly diagnosed with asymptomatic HT and then were diagnosed with PTC, the causal relationship between PTC and HT could not be established. Thus, our study mainly characterized the amount and TCR repertoire of T cells in PTC-W and PTC-WO and it is essential to clarify the relationship between PTC initiation and HT in the future study. Of course, due to small sample size in our study, more samples are still required to assess the TCR repertoire of patients with different clinical characteristics. Despite these provocative findings, there may be another possible limitation. Since we adopted TCRβ as a straightforward target to evaluate the TCR repertoire; however, it is possible that TCR repertoires may be expanded with distinct TCR α chains, when they share a common TCR β chain.

In light of the foregoing, although the number of TILs in PTC-W was significantly higher than that in PTC-WO, most TILs could react to thyroidal autoantigens but not tumor antigens in PTC-W. In contrast, although TILs rarely infiltrated into tumors in PTC-WO, these TILs were significantly different from T cells in adjacent normal tissue and thus were spatially confined to the tumor microenvironment, which suggested that these TILs might be reactive to tumor antigens and further clonally amplified in PTC-WO. TCR clones vary greatly between distinct tumor foci of the same PTC-W or PTC-WO and demonstrated the ITH of the T cell anti-tumor response. Therefore, multiregion biopsies are required to comprehensively identify the immune response in PTC-W or PTC-WO individually, which provide great insights into the local immune reaction of PTC-W and PTC-WO and increase our understanding of the intratumoral TCR repertoire in solid tumors. The identification of clonally expanded TCRs in distinct tumor regions could give a hint on selection of tumor-reactive TCRs, which could provide insights into the TILs-mediated adoptive cell therapy for thyroid malignancies.

## Data Availability Statement

Raw sequencing data were submitted to the Sequence Read Archive (BioProject No.PRJNA755141).

## Ethics Statement

The studies involving human participants were reviewed and approved by the Institutional Review Board of Beijing Cancer Hospital, China. The patients/participants provided their written informed consent to participate in this study. Written informed consent was obtained from the individual(s) for the publication of any potentially identifiable images or data included in this article.

## Author Contributions

BL and ZL directed this project. LC, CZ and DF designed the study. LC, HD performed experiments. CZ, LC and HH analyzed data and wrote the manuscript. All authors read and approved the final manuscript. All authors contributed to the article and approved the submitted version.

## Funding

This work was supported by grants from the National Key Research and Development Program of China (Grant No. 2019YFC0119200); the Natural Science Foundation of China (Grant No 82003246 to CZ, Grant No 81972880 to ZL); the Capital’s Funds for Health Improvement and Research (Grant No 2020-4-1028 to CZ); Open Project funded by Key laboratory of Carcinogenesis and Translational Research, Ministry of Education/Beijing (2022 Open Project-1); Cooperation Fund of Beijing Cancer Hospital and Beijing Institute for Cancer Research; Clinical Medicine Plus X - Young Scholars Project (PKU2022LCXQ036), Peking University, the Fundamental Research Funds for the Central Universities.

## Conflict of Interest

The authors declare that the research was conducted in the absence of any commercial or financial relationships that could be construed as a potential conflict of interest.

## Publisher’s Note

All claims expressed in this article are solely those of the authors and do not necessarily represent those of their affiliated organizations, or those of the publisher, the editors and the reviewers. Any product that may be evaluated in this article, or claim that may be made by its manufacturer, is not guaranteed or endorsed by the publisher.

## Supplementary Material

Supplementary Figure 1 Representative H&E and IHC staining images of CD3+ CD4+ and CD8+ T cells in PTC with and without HT (magnification ×400). **(A)** The representative images of P2 and P3. **(B)** The representative images of PH2 and PH3.

Supplementary Figure 2 The TCR Vβ repertoire usage of T cells in tumor (T) and normal (N) samples from PTC without HT.

Supplementary Figure 3 The TCR Vβ repertoire usage of T cells in tumor (T) and normal (N) samples from PTC with HT.

Supplementary Figure 4 The statistical results of CD3+ CD4+ and CD8+ T cells in PTC with and without HT. **(A)** The IHC staining scores of all patients. **(B)** The IHC staining scores in the subgroup of female patients. **(C)** The IHC staining scores in the subgroup of male patients.

## References

[1] Bray F Ferlay J Soerjomataram I Siegel RL Torre LA Jemal A. Global Cancer Statistics 2018: GLOBOCAN Estimates of Incidence and Mortality Worldwide for 36 Cancers in 185 Countries. CA: A Cancer J Clin (2018) 68(6):394–424. doi: 10.3322/caac.21492 30207593

[2] Lee JH Kim Y Choi JW Kim YS. The Association Between Papillary Thyroid Carcinoma and Histologically Proven Hashimoto's Thyroiditis: A Meta-Analysis. Eur J Endocrinol (2013) 168(3):343–9. doi: 10.1530/EJE-12-0903 23211578

[3] Ehlers M Schott M. Hashimoto's Thyroiditis and Papillary Thyroid Cancer: Are They Immunologically Linked? Trends Endocrinol Metab (2014) 25(12):656–64. doi: 10.1016/j.tem.2014.09.001 25306886

[4] Junttila MR de Sauvage FJ. Influence of Tumour Micro-Environment Heterogeneity on Therapeutic Response. Nature (2013) 501(7467):346–54. doi: 10.1038/nature12626 24048067

[5] Marusyk A Janiszewska M Polyak K. Intratumor Heterogeneity: The Rosetta Stone of Therapy Resistance. Cancer Cell (2020) 37(4):471–84. doi: 10.1016/j.ccell.2020.03.007 PMC7181408 32289271

[6] Waldman AD Fritz JM Lenardo MJ. A Guide to Cancer Immunotherapy: From T Cell Basic Science to Clinical Practice. Nat Rev Immunol (2020) 20(11):651–68. doi: 10.1038/s41577-020-0306-5 PMC7238960 32433532

[7] Tan Q Zhang C Yang W Liu Y Heyilimu P Feng D. Isolation of T Cell Receptor Specifically Reactive With Autologous Tumour Cells From Tumour-Infiltrating Lymphocytes and Construction of T Cell Receptor Engineered T Cells for Esophageal Squamous Cell Carcinoma. J Immunother Cancer (2019) 7(1):232. doi: 10.1186/s40425-019-0709-7 31462302 PMC6714102

[8] Lu Z Sheng J Zhang Y Deng J Li Y Lu A. Clonality Analysis of Multifocal Papillary Thyroid Carcinoma by Using Genetic Profiles. J Pathol (2016) 239(1):72–83. doi: 10.1002/path.4696 27071483 PMC5706659

[9] Gupta S Patel A Folstad A Fenton C Dinauer CA Tuttle RM. Infiltration of Differentiated Thyroid Carcinoma by Proliferating Lymphocytes is Associated With Improved Disease-Free Survival for Children and Young Adults. J Clin Endocrinol Metab (2001) 86(3):1346–54. doi: 10.1210/jc.86.3.1346 11238531

[10] Davis MM Bjorkman PJ. T-Cell Antigen Receptor Genes and T-Cell Recognition. Nature (1988) 334(6181):395–402. doi: 10.1038/334395a0 3043226

[11] Emerson RO Sherwood AM Rieder MJ Guenthoer J Williamson DW Carlson CS. High-Throughput Sequencing of T-Cell Receptors Reveals a Homogeneous Repertoire of Tumour-Infiltrating Lymphocytes in Ovarian Cancer. J Pathol (2013) 231(4):433–40. doi: 10.1002/path.4260 PMC5012191 24027095

[12] Padovan E Casorati G Dellabona P Meyer S Brockhaus M Lanzavecchia A. Expression of Two T Cell Receptor Alpha Chains: Dual Receptor T Cells. Science (1993) 262(5132):422–4. doi: 10.1126/science.8211163 8211163

[13] Robins HS Campregher PV Srivastava SK Wacher A Turtle CJ Kahsai O. Comprehensive Assessment of T-Cell Receptor Beta-Chain Diversity in Alphabeta T Cells. Blood (2009) 114(19):4099–107. doi: 10.1182/blood-2009-04-217604 PMC2774550 19706884

[14] Sherwood AM Emerson RO Scherer D Habermann N Buck K Staffa J. Tumor-Infiltrating Lymphocytes in Colorectal Tumors Display a Diversity of T Cell Receptor Sequences That Differ From the T Cells in Adjacent Mucosal Tissue. Cancer Immunol Immunother (2013) 62(9):1453–61. doi: 10.1007/s00262-013-1446-2 PMC5714653 23771160

[15] Zhang C Ding H Huang H Palashati H Miao Y Xiong H. TCR Repertoire Intratumor Heterogeneity of CD4(+) and CD8(+) T Cells in Centers and Margins of Localized Lung Adenocarcinomas. Int J Cancer (2019) 144(4):818–27. doi: 10.1002/ijc.31760 30151844

[16] Chen Z Zhang C Pan Y Xu R Xu C Chen Z. T Cell Receptor Beta-Chain Repertoire Analysis Reveals Intratumour Heterogeneity of Tumour-Infiltrating Lymphocytes in Oesophageal Squamous Cell Carcinoma. J Pathol (2016) 239(4):450–8. doi: 10.1002/path.4742 27171315

[17] Yousfi Monod M Giudicelli V Chaume D Lefranc MP. IMGT/JunctionAnalysis: The First Tool for the Analysis of the Immunoglobulin and T Cell Receptor Complex V-J and V-D-J JUNCTIONs. Bioinformatics (2004) 20 Suppl 1:i379–85. doi: 10.1093/bioinformatics/bth945 15262823

[18] Lu Z Zhang C Sheng J Shen J Liu B. T Cell Receptor Beta-Chain Repertoire Analysis Reveals the Association Between Neoantigens and Tumour-Infiltrating Lymphocytes in Multifocal Papillary Thyroid Carcinoma. Int J Cancer (2017) 141(2):377–82. doi: 10.1002/ijc.30743 28431188

[19] Zhang C Tan Q Li S Shen L Zhang J Liu Y. Induction of EBV Latent Membrane Protein-2A (LMP2A)-Specific T Cells and Construction of Individualized TCR-Engineered T Cells for EBV-Associated Malignancies. J immunother Cancer (2021) 9(7):e002516. doi: 10.1136/jitc-2021-002516 34210819 PMC8252876

[20] Rempala GA Seweryn M. Methods for Diversity and Overlap Analysis in T-Cell Receptor Populations. J Math Biol (2013) 67(6-7):1339–68. doi: 10.1007/s00285-012-0589-7 PMC3543521 23007599

[21] Wang Y Liu Y Chen L Chen Z Wang X Jiang R. T Cell Receptor Beta-Chain Profiling of Tumor Tissue, Peripheral Blood and Regional Lymph Nodes From Patients With Papillary Thyroid Carcinoma. Front Immunol (2021) 12:595355. doi: 10.3389/fimmu.2021.595355 33679738 PMC7930746

[22] Sun G Qiu L Cheng Z Pan W Qiu J Zou C. Association of the Characteristics of B- and T-Cell Repertoires With Papillary Thyroid Carcinoma. Oncol Lett (2018) 16(2):1584–92. doi: 10.3892/ol.2018.8800 PMC6036450 30008841

[23] Giordano TJ. Genomic Hallmarks of Thyroid Neoplasia. Annu Rev Pathol (2018) 13:141–62. doi: 10.1146/annurev-pathol-121808-102139 29083981

[24] Dagogo-Jack I Shaw AT. Tumour Heterogeneity and Resistance to Cancer Therapies. Nat Rev Clin Oncol (2018) 15(2):81–94. doi: 10.1038/nrclinonc.2017.166 29115304

[25] Vitale I Shema E Loi S Galluzzi L. Intratumoral Heterogeneity in Cancer Progression and Response to Immunotherapy. Nat Med (2021) 27(2):212–24. doi: 10.1038/s41591-021-01233-9 33574607

[26] Caturegli P De Remigis A Rose NR. Hashimoto Thyroiditis: Clinical and Diagnostic Criteria. Autoimmun Rev (2014) 13(4-5):391–7. doi: 10.1016/j.autrev.2014.01.007 24434360

[27] Luty J Ruckemann-Dziurdzinska K Witkowski JM Bryl E. Immunological Aspects of Autoimmune Thyroid Disease - Complex Interplay Between Cells and Cytokines. Cytokine (2019) 116:128–33. doi: 10.1016/j.cyto.2019.01.003 30711852

[28] Nicolson NG Brown TC Korah R Carling T. Immune Cell Infiltrate-Associated Dysregulation of DNA Repair Machinery may Predispose to Papillary Thyroid Carcinogenesis. Surgery (2020) 167(1):66–72. doi: 10.1016/j.surg.2019.02.024 31439400

