# Supplementary figures and images for: Clonal Distribution and Intratumor Heterogeneity of the TCR Repertoire in Papillary Thyroid Cancer With or Without Coexistent Hashimoto’s Thyroiditis
Source: Front Immunol. 2022 Jun 3;13:821601. doi: 10.3389/fimmu.2022.821601 (PMC9203861; doi:10.3389/fimmu.2022.821601)

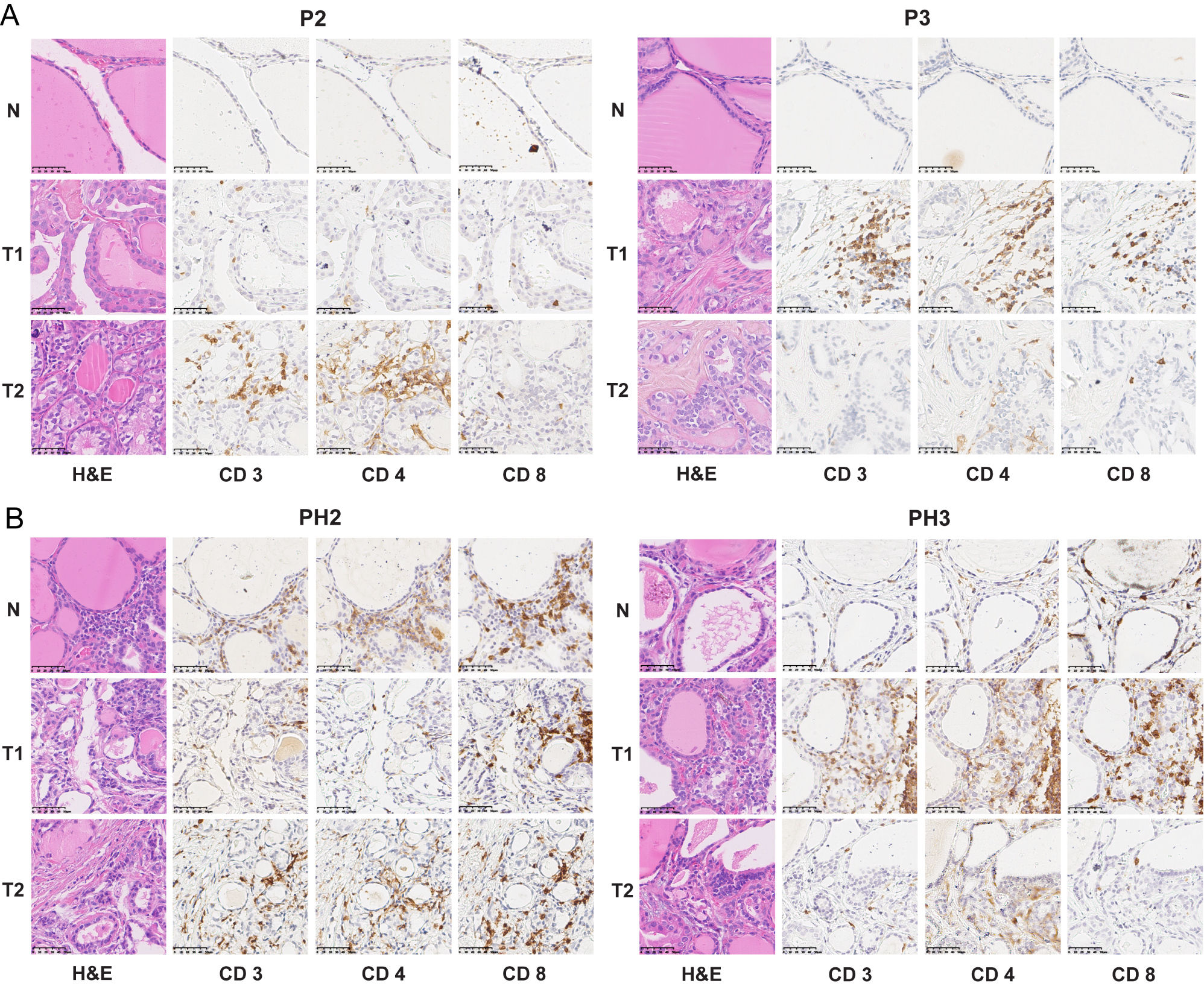

Supplement: Supplementary Figure 1 — Representative H&E and IHC staining images of CD3+ CD4+ and CD8+ T cells in PTC with and without HT (magnification ×400). (A) The representative images of P2 and P3. (B) The representative images of PH2 and PH3. [file Image_1.tif]

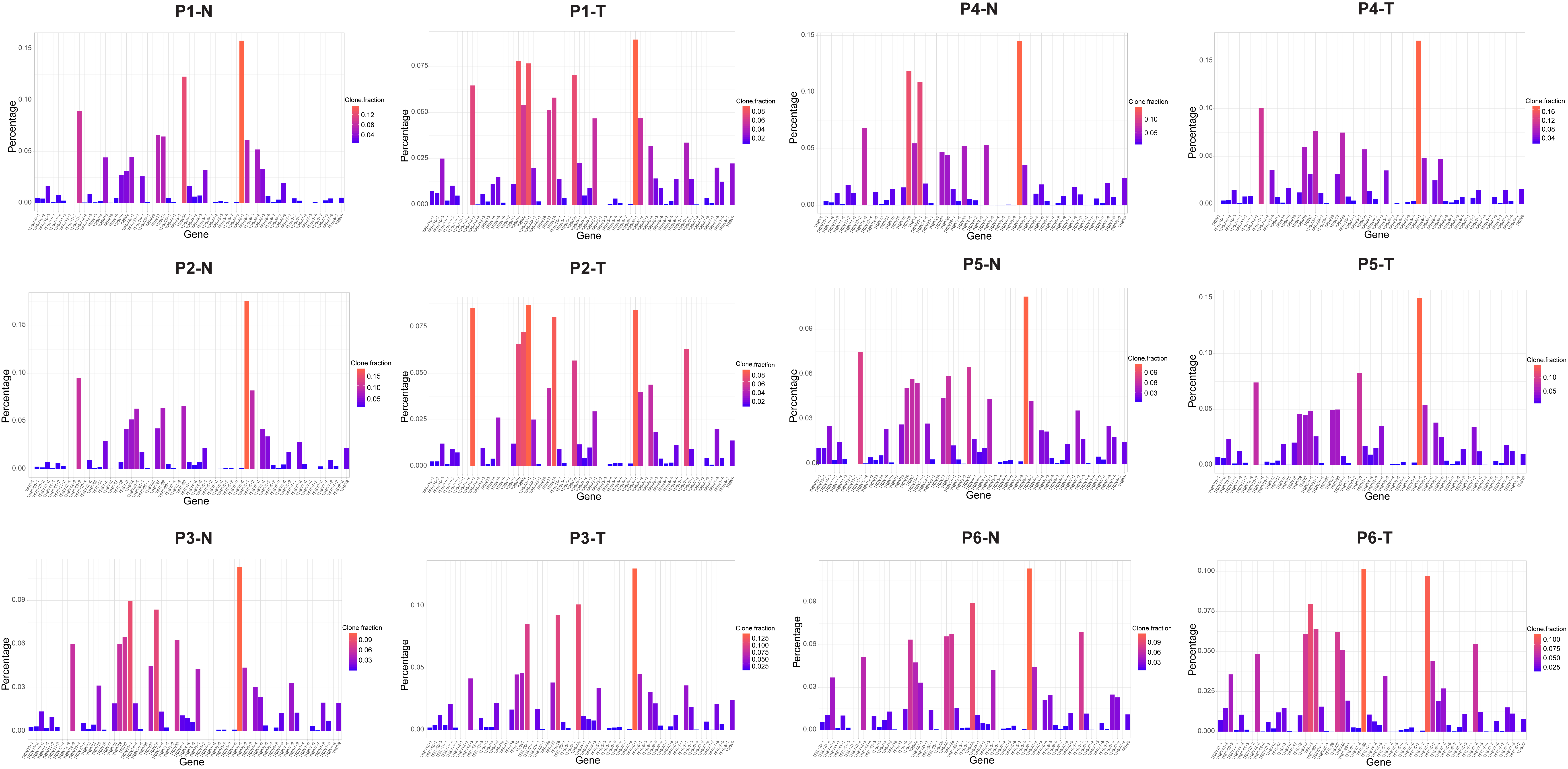

Supplement: Supplementary Figure 2 — The TCR Vβ repertoire usage of T cells in tumor (T) and normal (N) samples from PTC without HT. [file Image_2.tif]

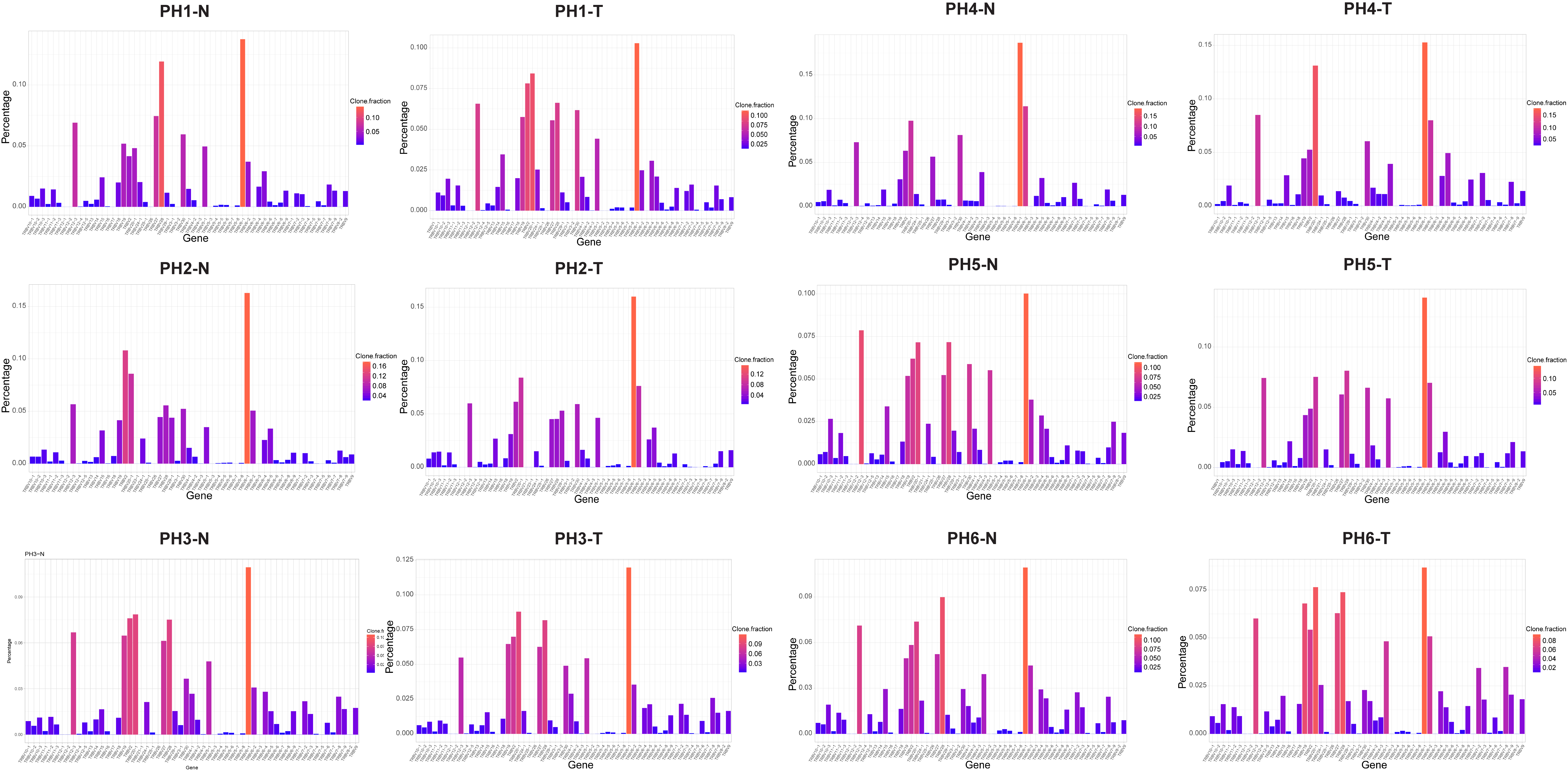

Supplement: Supplementary Figure 3 — The TCR Vβ repertoire usage of T cells in tumor (T) and normal (N) samples from PTC with HT. [file Image_3.tif]

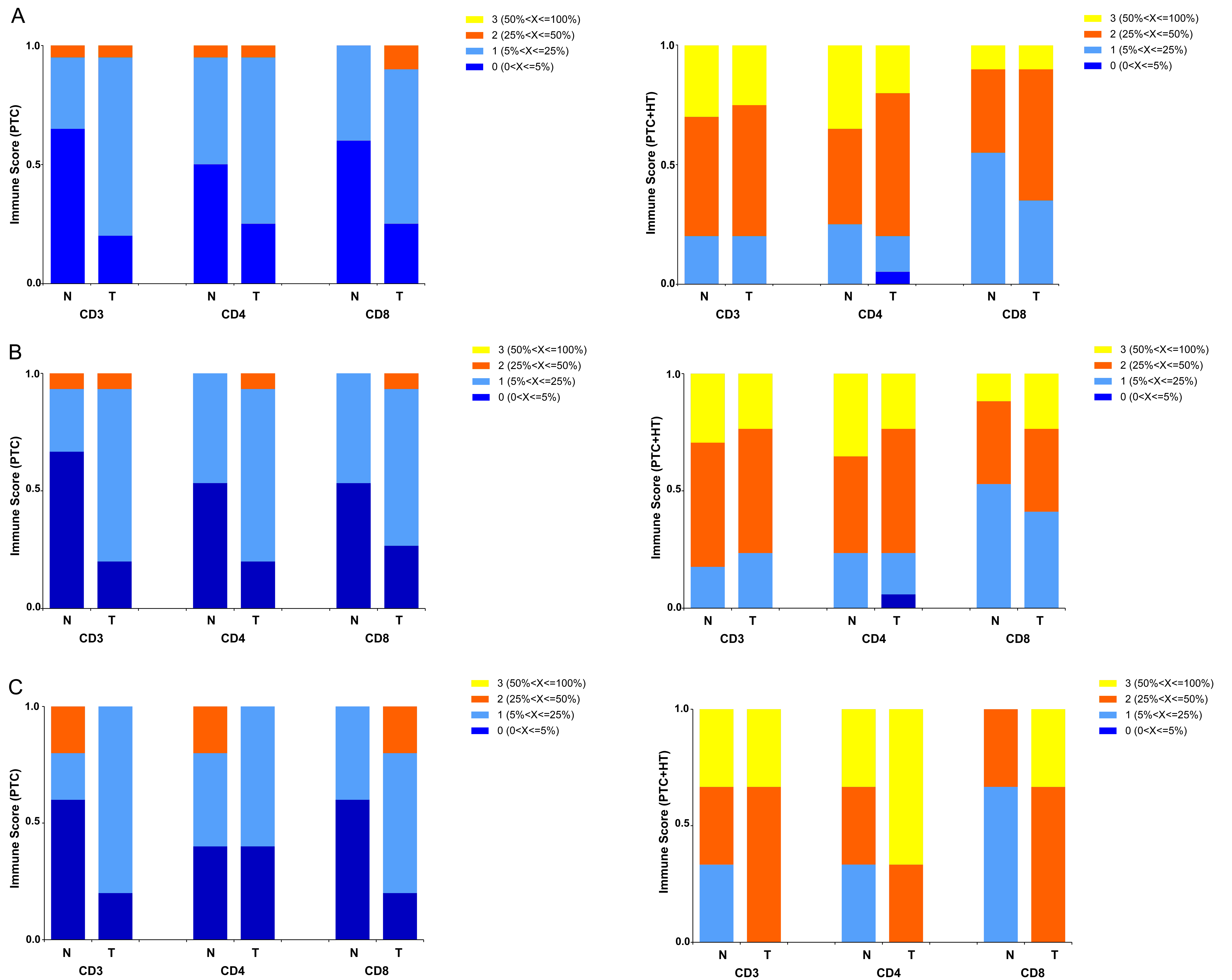

Supplement: Supplementary Figure 4 — The statistical results of CD3+ CD4+ and CD8+ T cells in PTC with and without HT. (A) The IHC staining scores of all patients. (B) The IHC staining scores in the subgroup of female patients. (C) The IHC staining scores in the subgroup of male patients. [file Image_4.tif]
