# Supplementary material for: Clonal Distribution and Intratumor Heterogeneity of the TCR Repertoire in Papillary Thyroid Cancer With or Without Coexistent Hashimoto’s Thyroiditis
Source: Front Immunol. 2022 Jun 3;13:821601. doi: 10.3389/fimmu.2022.821601 (PMC9203861; doi:10.3389/fimmu.2022.821601)
Supplement: Supplementary file 5 [file Table_1.docx]

Supplementary Table S1. Clinical characteristics of 40 patients with papillary thyroid cancer.

| **Characteristics** | **PTC-WO (n=20)** | **PTC-W (n=20)** | ***P* value** |
| --- | --- | --- | --- |
| Age (years) | 47.9 ± 2.8 | 42.0 ± 2.5 | > 0.05 |
| < 55 | 15 | 17 | > 0.05 |
| >= 55 | 5 | 3 |  |
| Sex (female/male) |  |  |  |
| male | 5 | 3 | > 0.05 |
| female | 15 | 17 |  |
| Tumor size (cm) | 1.1 (0.7, 1.5) | 1.0 (0.8, 1.5) | > 0.05 |
| < 1 | 9 | 8 | > 0.05 |
| >= 1 | 11 | 12 |  |
| TNM stage (I/II) |  |  |  |
| I | 18 | 19 | > 0.05 |
| II | 2 | 1 |  |
| TSH (mIU/L) | 1.7 ± 0.3 | 3.1 ± 0.5 | < 0.05 |
| Anti-TPOAb (IU/mL) | 19.5 ± 3.1 | 234.1 ± 46.3 | < 0.05 |
| Anti-TGAb (IU/mL) | 22.0 ± 4.1 | 327.2 ± 101.9 | < 0.05 |

*P values of continuous variables and categorical variables were calculated by t-test and chi-square test, respectively.
