# Supplementary material for: Clonal Distribution and Intratumor Heterogeneity of the TCR Repertoire in Papillary Thyroid Cancer With or Without Coexistent Hashimoto’s Thyroiditis
Source: Front Immunol. 2022 Jun 3;13:821601. doi: 10.3389/fimmu.2022.821601 (PMC9203861; doi:10.3389/fimmu.2022.821601)
Supplement: Supplementary file 6 [file Table_2.docx]

Supplementary Table S2. Demographic characteristics and IHC results in 40 patients with papillary thyroid cancer.

| Patient | Age | Gender | Concomitant with  Hashimoto's thyroiditis | IHC staining scores^a^ | | | | | | | | |
| --- | --- | --- | --- | --- | --- | --- | --- | --- | --- | --- | --- | --- |
|  |  |  |  | CD3_Normal | CD3_Tumor 1 | CD3_Tumor 2 | CD4_Normal | CD4_Tumor 1 | CD4_Tumor 2 | CD8_Normal | CD8_Tumor 1 | CD8_Tumor 2 |
| P1 | 67 | female | no | 0 | 2 | 2 | 0 | 2 | 2 | 0 | 1 | 1 |
| P2 | 46 | female | no | 1 | 1 | 2 | 1 | 1 | 2 | 1 | 1 | 1 |
| P3 | 46 | female | no | 1 | 1 | 1 | 1 | 1 | 1 | 0 | 0 | 0 |
| P4 | 52 | female | no | 0 | 1 | 0 | 1 | 1 | 0 | 1 | 1 | 0 |
| P5 | 25 | female | no | 0 | 0 | 1 | 0 | 0 | 1 | 0 | 1 | 1 |
| P6 | 54 | female | no | 1 | 0 | 0 | 1 | 0 | 0 | 1 | 1 | 0 |
| P7 | 45 | female | no | 0 | 0 | 0 | 0 | 0 | 0 | 0 | 1 | 0 |
| P8 | 31 | female | no | 1 | 1 | 0 | 1 | 0 | 1 | 1 | 1 | 0 |
| P9 | 62 | male | no | 0 | 1 | 1 | 0 | 0 | 0 | 0 | 1 | 0 |
| P10 | 49 | male | no | 1 | 2 | 0 | 2 | 1 | 0 | 1 | 2 | 1 |
| P11 | 50 | female | no | 0 | 1 | 1 | 0 | 1 | 0 | 0 | 1 | 1 |
| P12 | 31 | female | no | 2 | 2 | 1 | 1 | 1 | 1 | 1 | 2 | 2 |
| P13 | 35 | female | no | 0 | 1 | 2 | 0 | 1 | 2 | 0 | 0 | 1 |
| P14 | 51 | male | no | 0 | 0 | 0 | 1 | 0 | 0 | 1 | 0 | 0 |
| P15 | 57 | female | no | 0 | 0 | 0 | 0 | 0 | 0 | 0 | 0 | 0 |
| P16 | 32 | female | no | 0 | 2 | 1 | 0 | 2 | 0 | 0 | 0 | 1 |
| P17 | 40 | male | no | 0 | 2 | 0 | 0 | 2 | 0 | 0 | 1 | 0 |
| P18 | 61 | male | no | 1 | 2 | 0 | 1 | 2 | 0 | 0 | 1 | 1 |
| P19 | 70 | female | no | 0 | 1 | 1 | 1 | 0 | 2 | 1 | 0 | 0 |
| P20 | 54 | female | no | 0 | 0 | 1 | 0 | 0 | 1 | 0 | 1 | 1 |
| PH1 | 30 | female | yes | 1 | 2 | 3 | 1 | 2 | 3 | 1 | 2 | 2 |
| PH2 | 65 | female | yes | 1 | 1 | 1 | 1 | 1 | 2 | 1 | 1 | 2 |
| PH3 | 28 | female | yes | 2 | 1 | 2 | 2 | 1 | 2 | 1 | 0 | 2 |
| PH4 | 41 | female | yes | 3 | 3 | 2 | 3 | 3 | 1 | 3 | 2 | 1 |
| PH5 | 52 | female | yes | 2 | 2 | 2 | 2 | 3 | 3 | 2 | 2 | 3 |
| PH6 | 40 | female | yes | 3 | 2 | 2 | 2 | 2 | 2 | 2 | 2 | 2 |
| PH7 | 33 | female | yes | 2 | 2 | 2 | 1 | 1 | 1 | 1 | 0 | 1 |
| PH8 | 38 | female | yes | 1 | 2 | 2 | 1 | 2 | 2 | 1 | 2 | 1 |
| PH9 | 42 | female | yes | 2 | 3 | 1 | 2 | 2 | 1 | 2 | 2 | 2 |
| PH10 | 37 | male | yes | 2 | 2 | 2 | 3 | 2 | 3 | 2 | 2 | 1 |
| PH11 | 31 | female | yes | 2 | 2 | 1 | 2 | 1 | 3 | 1 | 1 | 2 |
| PH12 | 45 | female | yes | 2 | 3 | 2 | 3 | 2 | 3 | 2 | 1 | 2 |
| PH13 | 43 | female | yes | 2 | 1 | 1 | 2 | 1 | 0 | 1 | 1 | 1 |
| PH14 | 63 | female | yes | 3 | 2 | 2 | 3 | 3 | 2 | 2 | 2 | 3 |
| PH15 | 38 | female | yes | 2 | 1 | 2 | 3 | 2 | 3 | 1 | 2 | 1 |
| PH16 | 52 | female | yes | 3 | 3 | 2 | 3 | 2 | 3 | 3 | 1 | 3 |
| PH17 | 34 | female | yes | 2 | 3 | 2 | 2 | 2 | 2 | 1 | 2 | 2 |
| PH18 | 23 | female | yes | 3 | 3 | 2 | 3 | 1 | 3 | 2 | 1 | 1 |
| PH19 | 55 | male | yes | 1 | 2 | 2 | 1 | 3 | 2 | 1 | 2 | 2 |
| PH20 | 49 | male | yes | 3 | 3 | 2 | 2 | 3 | 2 | 1 | 3 | 3 |

a. The IHC staining was scored, according to the percentage of positive cells (0, ≤5%; 1, 6-25%; 2, 26-50%; 3, 51-75%; and 4, >75%).
