# Supplementary material for: Clonal Distribution and Intratumor Heterogeneity of the TCR Repertoire in Papillary Thyroid Cancer With or Without Coexistent Hashimoto’s Thyroiditis
Source: Front Immunol. 2022 Jun 3;13:821601. doi: 10.3389/fimmu.2022.821601 (PMC9203861; doi:10.3389/fimmu.2022.821601)
Supplement: Supplementary file 7 [file Table_3.docx]

Supplementary Table S3. TCRβ CDR3 sequencing metrics in twelve patients with papillary thyroid cancer.

| Patient | Concomitant with  Hashimoto's thyroiditis | Total productive  TCRβ sequences | | | Unique productive  TCRβ clones | | | Clonality | | |  |
| --- | --- | --- | --- | --- | --- | --- | --- | --- | --- | --- | --- |
|  |  |  |  |  |  |  |  |  |  |  |  |
|  |  | Normal | Tumor1 | Tumor2 | Normal | Tumor1 | Tumor2 | Normal | Tumor1 | Tumor2 |  |
| P1 | no | 122075 | 134557 | 104331 | 72175 | 81996 | 63924 | 0.137746143 | 0.16203687 | 0.191099886 |  |
| P2 | no | 103177 | 106230 | 118709 | 53373 | 64835 | 74522 | 0.198923146 | 0.221164227 | 0.20633456 |  |
| P3 | no | 249622 | 65237 | 64497 | 171059 | 38063 | 38368 | 0.117470908 | 0.228414126 | 0.204911572 |  |
| P4 | no | 23462 | 26158 | 18715 | 13397 | 15591 | 11807 | 0.301935833 | 0.307687664 | 0.310483691 |  |
| P5 | no | 148933 | 77352 | 139288 | 94222 | 45021 | 79709 | 0.145723437 | 0.206575554 | 0.192357794 |  |
| P6 | no | 71362 | 51064 | 53688 | 41954 | 28238 | 30820 | 0.266339718 | 0.273748785 | 0.281507102 |  |
| PH1 | yes | 128524 | 246178 | 244656 | 78356 | 173681 | 154303 | 0.12131053 | 0.141767076 | 0.160169289 |  |
| PH2 | yes | 53258 | 53346 | 41217 | 29963 | 31314 | 24341 | 0.216344136 | 0.212225245 | 0.225274388 |  |
| PH3 | yes | 156248 | 79645 | 122382 | 95471 | 45173 | 71600 | 0.142723863 | 0.18602132 | 0.182715000 |  |
| PH4 | yes | 27251 | 30012 | 10052 | 14433 | 17857 | 6382 | 0.263976827 | 0.262506947 | 0.320792936 |  |
| PH5 | yes | 371445 | 103481 | 215822 | 267594 | 63513 | 142005 | 0.096252849 | 0.139871327 | 0.113922432 |  |
| PH6 | yes | 111180 | 84388 | 85645 | 64202 | 50324 | 51582 | 0.182030878 | 0.193666424 | 0.194115508 |  |
